# Multiplex Platforms for the Identification of Respiratory Pathogens: Are They Useful in Pediatric Clinical Practice?

**DOI:** 10.3389/fcimb.2019.00196

**Published:** 2019-06-04

**Authors:** Susanna Esposito, Antonella Mencacci, Elio Cenci, Barbara Camilloni, Ettore Silvestri, Nicola Principi

**Affiliations:** ^1^Pediatric Clinic, Department of Surgical and Biomedical Sciences, Università degli Studi di Perugia, Perugia, Italy; ^2^Microbiology Unit, Department of Medicine, Università degli Studi di Perugia, Perugia, Italy; ^3^Università degli Studi di Milano, Milan, Italy

**Keywords:** multiplex platforms, PCR, respiratory pathogens, respiratory tract infection, influenza, respiratory syncytial virus

## Abstract

Respiratory tract infections (RTIs) are extremely common especially in the first year of life. Knowledge of the etiology of a RTI is essential to facilitate the appropriate management and the implementation of the most effective control measures. This perspective explains why laboratory methods that can identify pathogens in respiratory secretions have been developed over the course of many years. High-complexity multiplex panel assays that can simultaneously detect up to 20 viruses and up to four bacteria within a few hours have been marketed. However, are these platforms actually useful in pediatric clinical practice? In this manuscript, we showed that these platforms appear to be particularly important for epidemiological studies and clinical research. On the contrary, their routine use in pediatric clinical practice remains debatable. They can be used only in the hospital as they require specific equipment and laboratory technicians with considerable knowledge, training, and experience. Moreover, despite more sensitive and specific than other tests routinely used for respiratory pathogen identification, they do not offer significantly advantage for detection of the true etiology of a respiratory disease. Furthermore, knowledge of which virus is the cause of a respiratory disease is not useful from a therapeutic point of view unless influenza virus or respiratory syncytial virus are the infecting agents as effective drugs are available only for these pathogens. On the other hand, multiplex platforms can be justified in the presence of severe clinical manifestations, and in immunocompromised patients for whom specific treatment option can be available, particularly when they can be used simultaneously with platforms that allow identification of antimicrobial resistance to commonly used drugs. It is highly likely that these platforms, particularly those with high sensitivity and specificity and with low turnaround time, will become essential when new drugs effective and safe against most of the respiratory viruses will be available. Further studies on how to differentiate carriers from patients with true disease, as well as studies on the implications of coinfections and identification of antimicrobial resistance, are warranted.

## Introduction

Respiratory tract infections (RTIs) are extremely common especially in the first year of life (Everard, 2016). Most of these infections are due to one of the many respiratory viruses, mainly respiratory syncytial virus (RSV) (Griffiths et al., 2017), influenza virus (IV) (Antonova et al., 2012), and rhinovirus (RV) (Principi et al., 2014). However, parainfluenza virus (PIV) (Branche and Falsey, 2016), adenovirus (ADV) (Esposito et al., 2016b), human metapneumovirus (hMPV) (Principi and Esposito, 2014), bocavirus (BV) (Principi et al., 2015a), and enterovirus (EV) (Hellferscee et al., 2017) can also play a relevant role, particularly during epidemics. When bacteria are the cause, *Streptococcus pyogenes* is a common cause of pharyngitis (Paradise, 1992), and *Streptococcus pneumoniae* is a typical cause of lower RTIs (Esposito and Principi, 2012). In some cases, coinfections with two or more viruses (Scotta et al., 2016) or with viruses and bacteria (Brealey et al., 2015) can occur.

For many years, it was thought that knowledge of the etiology of a respiratory infection was essential to facilitate the appropriate management and the implementation of the most effective control measures. It was presupposed that evidence showing that a given viral pathogen was the cause of a respiratory infection could reduce the prescription of further diagnostic tests and the use of antibiotics. Theoretically, clinician uncertainty and the anxiety of patients and their family members could also be reduced (Gill et al., 2017). This perspective explains why laboratory methods that can identify pathogens in respiratory secretions have been developed over the course of many years. Initially, cell cultures, immunofluorescence assays, and rapid antigen direct tests were used. These tests were mainly used for virus identification due to the higher frequency of viral RTIs. However, none of these tests were considered completely satisfactory for clinical use. Although cell cultures exhibited a high specificity and good sensitivity, they were very expensive and had a long turnaround time (Leland and Ginocchio, 2007; Gharabaghi et al., 2011). Immunofluorescence assays achieved moderate sensitivity, identified no more than eight viruses, and sometimes required a long turnaround time (Ginocchio and McAdam, 2011). Finally, rapid antigen direct tests, although able to provide results in few minutes with high specificity, were available only for RSV, IV, and adenovirus and have low sensitivity (Gharabaghi et al., 2011; Ginocchio and McAdam, 2011).

These problems have been overcome, at least in part, in recent years, when methods based on nucleic acid amplification became available. Such methods exhibit enhanced sensitivity and specificity, and they can detect a broad range of pathogens within an acceptable turnaround time. Single polymerase chain reactions (PCRs) for all the known respiratory viruses and several multiplex platforms using PCR and methods for nucleic acid amplification for the simultaneous detection of two or more viruses have been developed (Hanson and Couturier, 2016). High-complexity multiplex panel assays that can simultaneously detect up to 20 viruses (Mahony et al., 2007), 18 viruses and two or three atypical bacteria (Gonsalves et al., 2019), 18 viruses and four bacteria (Beckmann and Hirsch, 2016) and a total of 33 pathogens including 12 bacteria (Fast Track Diagnostic, 2018) within a few hours have been developed. However, not all the bacteria that play a relevant role in the determination of respiratory infections are systematically included. Some of these multiplex platforms have been marketed and largely evaluated in clinical practice in patients admitted to the emergency departments, hospital wards and intensive care units. Despite these evaluations, the actual role of these diagnostic measures is not precisely defined. In particular, it has not yet been established whether the availability of a laboratory measure able to identify several potential etiologic agents of a respiratory infection offers real advantages in term of diagnostic accuracy, choice of appropriate therapy and reduction of the social and economic problems strictly associated with pediatric respiratory diseases. Moreover, in the majority of the cases they don't allow the identification of antimicrobial resistance to commonly used drugs. In this paper, what can be derived from the presently available studies in this regard is discussed.

## What is the Efficiency of Multiplex Platforms in the Identification of Pathogens?

Multiplex platforms based on molecular methods can be used only in the hospital, as they require specific equipment and laboratory technicians with considerable knowledge, training, and experience (Beckmann and Hirsch, 2016; Esposito et al., 2016a; Biomerieux, 2018; Fast Track Diagnostic, 2018). Moreover, these platforms have a turnaround time that is significantly shorter than that of culture but generally much longer than that of rapid tests as they take some hours to give reliable results. This can be a limitation in the emergency department or in the intensive care unit, where many patients require immediate diagnostic and therapeutic decisions (Beckmann and Hirsch, 2016; Esposito et al., 2016a; Biomerieux, 2018; Fast Track Diagnostic, 2018). Only the most recently developed platforms, such as the BioFire® FilmArray® Respiratory Panel 2, have an acceptable turnaround time of about 1 h, not much longer than a rapid test (Biofire, 2018). Finally, the number of samples that can be processed per run can significantly vary from assay to assay. In some cases, such in the case of the already cited BioFire® assay, only one sample could be processed per run, while most platforms have higher sample throughput (up to 96 samples) (Chan et al., 2018). This can be a problem during epidemics when several patients have to be tested simultaneously.

Multiplex assays are significantly more sensitive and specific compared with rapid immunochromatographic tests and immunofluorescence assays; however, as multiplex assays detect both viable and non-viable viruses and bacteria, they can lead to debatable results (Beckmann and Hirsch, 2016; Esposito et al., 2016a; Biomerieux, 2018; Fast Track Diagnostic, 2018). Generally, the presently developed and marketed multiplex assays exhibit comparable performance with regards to sensitivities and specificities, and detection of coinfections. A previous comparison of the (Luminex, 2018) Nx TAG RPP assay, BioFire Film Array Respiratory Panel (FA-RP) (Biomerieux, 2018), RespiFinder22 (RF22) (Beckmann and Hirsch, 2016), and the (Luminex, 2018) RVP FAST Assay v2 (Esposito et al., 2016a) in terms of the ability to detect common pathogens revealed that the discordance was lower than 10%, although the turnaround time, workflow simplicity and risk of contamination were lower for the (Luminex, 2018) Nx TAG RPP (Chen et al., 2016; Tang et al., 2016). A systematic review and meta-analysis (Huang et al., 2018) of studies on the accuracy of FA-RP, Nanosphere Verigene RV+ test (Hologic, 2018; Luminex, 2018) Gen-Probe Prodesse assays (Hologic, 2018) in the detection of IV A, IV B virus, RSV, hMPV, and AV showed that all of these assays had high diagnostic accuracy, with an area under the receiver operating characteristic curve (AUROC) equal to or >0.98 for all tested viruses. The only exception was adenovirus, for which the AUROC was 0.89. Finally, a similarly high accuracy was demonstrated for Anyplex II RV16, AdvanSure RV, and Real-Q RV (Yun et al., 2018).

The Allplex Respiratory Panels (Allplex; Seegene, Republic of Korea), a recently released one-step real-time reverse transcription-PCR method for the simultaneous detection of multiple pathogens, has been demonstrated to be a rapid and accurate method for detecting respiratory viruses, particularly in case of multiple viral infections (Lee and Lee, 2019). A study comparing Allplex RP1 assay with ProdesseProFlu+ and ProFAST+ (Hologic, Madison, WI, USA), and GeneXpert Flu/RSV XC (Cepheid, USA) for IV and RSV detection, found accuracy of 95, 91, and 96% and sensitivity of 94, 88, and 95%, respectively. The three assays showed a 100% specificity and positive predictive value (PPV), while the negative predictive values (NPV) were 84, 73, and 86% for Allplex RP1, Prodesse and GeneXpert, respectively (Gimferrer et al., 2018).

In general, the accuracy of multiplex platforms in identifying respiratory pathogens is similar to those of single PCR, although in some cases, a slightly lower efficiency has been shown. For example, when the Luminex (2018) Nx TAG RPP was compared with single PCR, it was shown that the multiplex assay had a lower sensitivity against CV HKU1 and CV O43 and was oversensitive for several other viruses, leading to several false-positive results. The worst accuracy was found for hMPV, for which the PPV was <50% (Chen et al., 2016). Similarly, compared with standard PCR assays, the sensitivity of TaqMan Array Card was 54, 56, and 75% for ADV, PIV-1 and−2, respectively, and 82–95% for the other tested viruses. Assay specificity was 99%, and coefficients of variation for virus controls ranged from 1.5 to 4.5% (Weinberg et al., 2013).

However, as previously reported, not all the bacterial pathogens that cause respiratory infections are systematically included in the available multiplex platforms. This is a relevant limitation in clinical practice for most of these assays as it is the lack of information regarding antimicrobial resistance to the most commonly used anti-infective drugs.

## Do Multiplex Platforms Identify the True Etiology of a Respiratory Disease?

Evidence showing that a virus is present in the respiratory secretions of a patient with an RTI does not necessarily mean that that particular infectious agent is the cause of the disease. A virus can be the etiologic agent, but viruses can also be asymptomatically carried or shed for several weeks after an infection that has been cured. Therefore, viruses can be identified in the asymptomatic incubation period without having an actual role in the disease. Contrary to what was thought some years ago, viruses can asymptomatically colonize the airways. Both children admitted to the hospital for non-respiratory diseases and healthy children attending day care were shown to be carriers of at least one respiratory virus in ~30% of cases, although the viral load was generally lower in carriers than in symptomatic subjects (Jansen et al., 2011; Moe et al., 2016). Notably, the rate of asymptomatic colonization varies significantly from virus to virus. The detection of IV, RSV, hMPV, and PIV is generally indicative of disease, as the frequency of asymptomatic carriage of these agents is relatively uncommon. In contrast, the detection of certain other viruses raises many doubts due to the high frequency with which these viruses can be identified in asymptomatic children (Rhedin et al., 2014).

Despite the capacity to cause upper and lower RTIs and to trigger asthma and chronic obstructive pulmonary disease exacerbations (Esposito et al., 2012b; Parker et al., 2014; Principi et al., 2014), RV is the virus that is most frequently found in asymptomatic patients. Principi et al. (2015b) studied 88 healthy children <2 years old who were followed with weekly nasopharyngeal samples during the months of the winter season of 2013–2014. A total of 1,408 nasopharyngeal samples were obtained, and 326 samples tested positive for RV (23.1%). Of these, 209 (64.1%) were not associated with respiratory symptoms, suggesting asymptomatic colonization.

Carriage is common also for BV (Chow and Esper, 2009), which can be detected in the respiratory secretions of healthy children with the same frequency as that observed in patients with RTI (von Linstow et al., 2008). Similar findings have been reported for ADV, which has been detected in the nasopharynx of healthy children in up to 11% of cases (Colvin et al., 2012; Rhedin et al., 2014). Pathogens that are asymptomatically carried are also the pathogens that can be most commonly detected with the most recent multiplex platforms. The carriage of *Mycoplasma pneumoniae* (*Mp*) has been identified in many asymptomatic children, although the prevalence varied according to the site of the study [21% in Denmark (Spuesens et al., 2013), and 56% in the USA (Wood et al., 2013)] and the period of respiratory sample collection [3% in the spring, and 58% in the summer (Colvin et al., 2012)]. Similarly, a certain percentage of healthy children (4–6%) has been shown to test positive for *Chlamydophila pneumoniae* (*Cp*) (Emre et al., 1994; Block et al., 1997; Falck et al., 1997).

Long-term viral shedding after a previous infection can further render the results of multiplex platforms poorly effective in identifying the etiology of a disease. However, there are significant differences in long-term shedding among the various infectious agents. Generally, those agents that are uncommonly carried are the same agents that are shed only for a few days, and the opposite trend applies for those agents that are frequently asymptomatically carried. Although certain influenza patients can shed the virus up to 8 days, shedding typically peaks on day 1 after the onset of symptoms (Ip et al., 2017). Although there are exceptions (Munywoki et al., 2015), RSV shedding has been estimated to last between 3.4 and 7.4 days (Hall et al., 1976; Okiro et al., 2010). In contrast, RV and BC are generally shed for a longer time. In a study involving 46 children aged 6–36 months with bocavirus infection, it was shown that in 22% of cases, the virus persisted in the respiratory secretions for more than 30 days, despite the rapid disappearance of clinical manifestations (Wagner et al., 2016). The same prolonged shedding has been reported for RV (Loeffelholz et al., 2014), although there are differences according to the type of the infecting strain (Daleno et al., 2013). Long-lasting persistence in the respiratory secretions after acute infection has been demonstrated for ADV (Kalu et al., 2010) and atypical bacteria. The persistence of *Mp* DNA in the throat is common, with a median carriage time of 7 weeks after the disease onset (range 2 days−7 months) (Nilsson et al., 2008). *Cp* can be detected for months, as this pathogen temporarily interrupts its replication cycle when exposed to antimicrobials but later resumes replication, leading to the generation of infectious particles (Panzetta et al., 2018).

Finally, viruses can be detected during the incubation period without playing any role in the determination of the actual disease. The incubation period for viral respiratory infections is generally short. It has been calculated (Lessler et al., 2009) that this period lasts 5.6 days (95% confidence interval [CI], 4.8–6.3) for ADV, 3.2 days (95% CI, 2.8–3.7) for human coronavirus, 1.4 days (95% CI, 1.3–1.5) for IV A, 0.6 days (95% CI, 0.5–0.6) for IV B, 2.6 days (95% CI, 2.1–3.1) for PIV, 4.4 days (95% CI, 3.9–4.9) for RSV, and 1.9 days (95% CI, 1.4–2.4) for RV. However, during this period, these viruses are detectable in the respiratory secretions and can lead to diagnostic mistakes.

Defining the etiology of disease is further complicated by the detection of coinfections, as it is practically impossible to establish which agent is the true causative agent. Unfortunately, coinfections are common. In a study involving 592 children with radiographically confirmed CAP, viral coinfections were demonstrated in 117 cases (19.7% of the enrolled patients and 26.9% of those with viral infections). Similar findings were reported when viral-bacterial coinfections were studied (Nolan et al., 2018). These findings are not surprising, as a previous or concurrent viral RTI can favor the development of a secondary bacterial coinfection throughout the airway. Augmented bacterial adherence and colonization, dysregulation of the innate and adaptive immune response, immunosuppression, the release of bacteria from biofilms, and alteration of the microbiome are mechanisms through which viruses can favor bacterial superinfection (Bakaletz, 2017). In conclusion, multiplex platforms, despite significantly increasing the possibility to detect which pathogens are present in the respiratory secretions of a child with a respiratory infection, do not offer any advantage in comparison to tradition diagnostic tests regarding the identification of the true etiologic agent of the disease.

## Can Multiplex Platforms Significantly Contribute to the Prescription of the Most Appropriate Therapy?

When multiplex assays are used, the benefits of determining which infectious agent(s) is (are) potentially responsible for an RTI are strongly limited by the low number of drugs that are active against the respiratory targets that are currently available on these diagnostic platforms. Presently, only drugs against influenza virus, RSV and atypical bacteria are licensed. Furthermore, it is debated that these drugs should be used in all the subjects suffering from infections due to sensitive agents. The systematic use of neuraminidase inhibitors (European Center for Disease Prevention and Control, 2018) and baloxavir marboxil (Hayden et al., 2018) is not recommended for all the cases of influenza because influenza is frequently a mild disease, and the advantage of drug administration is limited to a marginal reduction in the disease duration. Consequently, the use of these drugs is reserved only for extremely severe cases, although their true efficacy in these cases has not been definitively demonstrated (Lessler et al., 2009; European Center for Disease Prevention and Control, 2018).

RSV infection can be treated with ribavirin, which is the only licensed drug for the treatment of this virus. However, ribavirin is difficult to use, costly, and teratogenic, and there is weak evidence for its efficacy. Ribavirin is typically used in severe cases that occur in immunocompromised subjects (Brendish and Clark, 2017). Although several new drugs against RSV are in development, and it is likely that in next few years some of them will be licensed for universal use, the present treatment of RSV infection remains based on supporting measures, such as hydration and O_2_ administration (Xing and Proesmans, 2019).

Antibiotics that are effective *in vitro* against atypical bacteria are generally recommended and largely used when these pathogens are the suspected or demonstrated cause of an RTI (Esposito et al., 2006, 2012a; Kohlhoff and Hammerschlag, 2015). However, the actual relevance of these drugs in clinical practice is debated, and there is evidence that seems to suggest that they are only slightly effective or not effective (Spuesens et al., 2014; Gardiner et al., 2015). In children, only macrolides can be used against *Mp* and *Cp*, as other drugs that are effective against atypical bacteria cannot be prescribed to these patients, particularly the youngest. The administration of tetracyclines and chloramphenicol can lead to severe adverse events; ketolides and streptogramins have limited use in pediatrics; and fluoroquinolones are not licensed for use in subjects <18 years of age (Principi and Esposito, 2001). However, many studies that have compared the clinical course of *Mp* and *Cp* infections in children treated with macrolides or other drugs that are ineffective against these pathogens have reported no difference between the two groups, suggesting that macrolides are useless (Principi and Esposito, 2001). In addition, macrolides should be used only when their use is presumed to be effective in reducing the abuse of antibiotics and the emergence of resistant strains (Principi and Esposito, 2013).

Finally, despite its high risk of nephrotoxicity, cidofovir, which is a drug licensed for the treatment of cytomegalovirus (CMV) retinitis in HIV-infected patients, has been used to treat ADV infections in immunocompromised subjects (Ganapathi et al., 2016). A retrospective evaluation including 16 children showed that in 10/16 cases, viral clearance and clinical response were achieved. However, four patients expired despite the viral clearance, and one of these deaths was directly ascribed to the ADV infection. However, cidofovir is not licensed for the treatment of ADV; this drug is reserved only for very severe cases occurring in immunocompromised children and cannot be considered as a potential solution to treat mild-to-moderate cases of respiratory disease in otherwise healthy children (Ganapathi et al., 2016).

## Can Multiplex Platforms Reduce the Medical and Socioeconomic Burden Associated With Respiratory Problems?

A recent prospective study including 284 children and 232 adults with RTI, aimed to determine antibiotic misuse, showed that viral infection was more common in children than in adults, while antibiotic overuse occurred both in children (37%) and, at a significantly higher level, in adults (83%). The study highlights the needing for effective interventions to decrease antibiotic overuse in RTI patients of all ages (van Houten et al., 2019).

It has been proposed that the use of tests that can identify viruses reduce the use of antibiotics, the prescription of other diagnostic measures, the risk of hospital acquisition among other patients and the length of stay in the pediatric clinical setting. The advantages of these tests from a medical, social, and economic point of view should therefore be enormous. However, the results of studies on multiplex assays are few and conflicting. When these tests were used in pediatric patients admitted to the intensive care unit, no advantage in antibiotic prescriptions was demonstrated (Byington et al., 2002). In contrast, a retrospective evaluation of the use of these tests in pediatric inpatients showed that a positive test result was associated with a decreased length of stay in the hospital and a shorter duration of antibiotics, at least in patients with common respiratory diagnoses (Schulert et al., 2013). However, in Wishaupt et al. (2011) the availability of results within 12–36 h did not lead to any advantage in terms of hospital admissions, length of hospital stay or the duration of antibiotic use when antibiotic treatment had been initiated. A more recent retrospective study in which a multiplex assay was tested in children admitted to the emergency department prior to admission or within the first 2 days of hospitalization revealed that patients for whom the results of the test were available were less likely to receive antibiotics for ≥2 days; moreover, these patients were more likely to be in isolation for ≥2 days compared with the controls (Subramony et al., 2016).

Finally, a recent study conducted in special high-risk settings such as hematology and oncology units, suggests that the diagnoses of asymptomatic virus infections, such as RSV and influenza, can be useful to lower the risk of hospital acquisition. The screening program proved useful for identifying asymptomatically infected patients with viral shedding, thus reducing the risk of transmission and potential nosocomial clusters of RSV and influenza virus on hemato-oncological wards (Baier et al., 2018).

On the other hand, less than satisfactory results were also reported in studies that evaluated the impact of rapid tests or immunofluorescence assays, although marketed preparations have been shown to exhibit sensitivity and specific higher than 90% (Vos et al., 2019). A Cochrane Review (Doan et al., 2014) that analyzed four studies regarding the impact of rapid viral diagnosis in children admitted to the Emergency Department reported that the only advantage of the rapid diagnosis was a lower rate of chest radiography (relative risk [RR], 0.77; 95% CI, 0.65–0.91). No effect on the length of the visit or blood or urine testing was demonstrated. Moreover, a trend toward decreased antibiotic use was demonstrated, but the difference between the tested and untested children was not significant. The knowledge that a bronchiolitis case was associated with the detection of RSV in the nasopharyngeal secretions did not lead to any advantage. Children with positive results from the rapid test received similar blood tests and radiological examinations. Moreover, although the children who tested positive had a more severe disease, virologic testing for RSV did not identify children at risk. Globally, the rapid test for RSV is considered useless, and this attitude explains why the test is not recommended by several experts (Ralston et al., 2014). The only rapid test for a single virus that seems to positively impact physician attitudes and have a satisfactory effect on antibiotic consumption the clinical course of disease is the rapid test for influenza. Several years ago, Esposito et al. (2003) showed that children with a positive rapid influenza test were significantly less likely than those with a negative test or no test to undergo routine blood examinations (2.3% vs. 14.5% and 15.0%; *p* = 0.045 and *p* = 0.038) or receive antibiotics (32.6% vs. 64.8% and 61.8%; *p* < 0.0001 and *p* = 0.0003). Children with positive tests also tended to have a lower incidence (although not significant) of chest radiographs (4.6% vs. 11.5% and 11.7%) and a lower likelihood of admission (0% vs. 4.6% and 5.8%). Similar results were reported more recently by Cantais et al. (2018), who showed that the diagnosis of influenza was followed by a 47.9% reduction in blood puncture, a 69.0% reduction in chest X-rays, a 77.8% reduction in lumbar puncture, a 79.2% reduction in urine culture, a 70.1% reduction in antibiotic treatments, and a 25.0% reduction in hospital stay; altogether, these reductions resulted in a reduction of medical costs estimated to be >€69,000 per season.

Finally, no studies have evaluated the impact of multiplex platforms on the expectations of patient and parents. Generally, laboratory tests are requested because they are considered to be effective measures to improve the judgement of clinicians. However, the diagnosis of a viral disease by a laboratory test can be interpreted as a simplification of a more complex clinical problem. Bronchiolitis due to RSV can be very severe, and its inclusion among a viral diagnosis may lead to parental dissatisfaction, especially given that no specific therapies are available for RSV, and only supportive measures are prescribed (Cabral et al., 2014). Moreover, no definitive information is available on the impact of multiplex assays on clinician anxiety. All these data are needed for a more complete evaluation of the impact of multiplex assays in clinical practice.

## Conclusions

Multiplex platforms for the identification of respiratory viruses and atypical bacteria allow for the identification of most of the infectious agents that cause respiratory infections in infants and children. It is highly likely that these platforms can be particularly important for studies specifically planned to evaluate epidemiology of respiratory pathogens and clinical research. On the contrary, their routine use in pediatric clinical practice remains debatable. They cannot be used in the community where most of the pediatric respiratory diseases are diagnosed. Moreover, they cannot allow to overcome the limitation of the traditional diagnostic tests for respiratory pathogens as they do not differentiate carriage from infection, do not seem to influence therapy as effective drugs are available only for IV and RSV, and do not seem to significantly impact of the socioeconomic problems strictly related to pediatric respiratory infections. They seem, however, justified in the presence of severe clinical manifestations, and in immunocompromised patients for whom specific treatment option can be available, particularly when they can be used simultaneously with platforms that allow identification of antimicrobial resistance to commonly used drugs. It is highly likely that these platforms, particularly those with high sensitivity and specificity and with low turnaround time, will become essential when new drugs effective and safe against most of the respiratory viruses will be available.

## Author Contributions

SE wrote the first draft of the manuscript. AM, EC, and BC gave a substantial scientific contribution. ES performed the literature review. NP co-wrote the first draft of the manuscript and supervised the project. All authors approved the final submitted version of the manuscript.

### Conflict of Interest Statement

The authors declare that the research was conducted in the absence of any commercial or financial relationships that could be construed as a potential conflict of interest.
